# Multiple sclerosis in Iran: An epidemiological update with focus on air pollution debate

**Published:** 2021-02-17

**Authors:** Masoud Amiri

**Affiliations:** ^1^Department of Epidemiology, Erasmus Medical Center, Rotterdam, The Netherlands; ^2^Department of Child and Adolescent Psychiatry/Psychology, Erasmus Medical Center, Rotterdam, The Netherlands; ^3^Generation R Study Group, Erasmus Medical Center, Rotterdam, The Netherlands

**Keywords:** multiple sclerosis, air pollution, epidemiology, Iran

## Abstract

**Background:**

Multiple sclerosis (MS), as the most common neurologic disorder of the central nervous system, with growing incidence and prevalence worldwide and in the Middle East. This article aimed to find out the potential relationship between MS and air pollution in Iran.

**Methods:**

By assessing the published articles on MS and air pollution in Iran, the situation of MS as well as air/soil pollution in Iran was clarified. Then, studies on air pollution and its potential effect on Iranian MS patients were checked until 2020.

**Results:**

The MS prevalence is distributed across Iran Provinces with highest rates in Isfahan, located in the center of Iran. The higher rates of MS in Isfahan and Tehran (the Metropolitan) might be due to industrial pollution of these cities, but this hypothesis is not true for non-industrial provinces. Based on the published atlas of MS in Iran, it seems that there is a high-risk “belt” from northwest to southeast.

**Conclusion:**

There are many risk factors of MS in Iran including age, gender, Vitamin D deficiency, smoking, and air pollution. The potential main risk factor of MS might be air pollution considering Isfahan and Tehran provinces. However, Chahar Mahal and Bakhtiary Province, with non-industrial nature, has the second highest MS rates which does not follow this hypothesis.

**Relevance for patients:**

By finding the air pollution as the main potential risk factor of MS in big provinces including Isfahan and Tehran, its effect of this factor can also be considered during diagnosis and treatment.

## 1. Introduction

Multiple sclerosis (MS) can be considered as the most common neurologic disorder of the central nervous system (CNS) which is diagnosed by inflammation, demyelination of neurons, and damages to the CNS [1]. MS is the second most common disability among younger adults after trauma [2]. There is a wide variation in the prevalence of MS in different geographical regions [3]. MS can indeed affect on at least more than 2 million people worldwide [4]. Most of the time, this disease has an ascendant and recurring trend [1]. The appearance and symptoms of this disease are suddenly occurring and may cause decease of the patient over several weeks to months, with usual age range of 17-50 years [1]; but with the late onset of MS (after age 50) [5] with age ranges from 27 to 80 years, 31-95 years [6]. In addition, MS patients with childhood-onset of MS may take longer to reach states of irreversible disability; however, they do so at a younger age compared to patients with adult-onset MS [7].

MS has even some effects on the personality of individuals: while MS patients with disease duration less than or equal 1 year have more likely type A personality, MS patients with more disease duration may have more likely type B personality [8]. In general, there is no treatment for MS [9].

There are potential limitations regarding interoperation of the researches; for example, it is not longitudinal in nature, so one cannot assess patient exposure throughout their lifetime and influence on MS risk [10]. Moreover, due to high number of patients, investigation of other environmental confounders (smoke habits, working activities, Vitamin D level, etc.) is not possible [10]. Furthermore, MS patients often have numerous complicated needs and, consequently, require a broad range of health services; however, there are some evidences indicating that patients’ needs are only partially met [11]. Despite all attempts of governmental and nongovernmental organizations for health-care delivery to MS patients, these services cannot satisfy all needs of the patients [11].

In 1980, Kurtzke hypothesized north-south latitude hypothesis, that in higher latitude, the prevalence of MS is larger; he classified world geographical areas into three regions based on MS prevalence: (1) High with more than 30/100,000, (2) intermediate between 5 and 25/100,000), and (3) low risk with less than 5/100,000. He mentioned that Middle-Eastern countries can be generally classified as low-risk areas [12]. However, MS is now a recurrent neurological disease with growing incidence and prevalence worldwide and in the Middle East [13-15]. The prevalence of 20 (1995) [16] and 39 (2005) [17] in Jordan, 14.77 (2000) [18], 85 (2013) [16] and 105 (2020) [19] in Kuwait, 25 (1998) [16], 40 (2008) [20], 31-55 (2013) [16] and 41 (2020) [19] in Saudi Arabia, 65 (2010) [16] and 98 (2020) [19] in Qatar, 42 in Cyprus (2010) [16], 51 (2011) [16] and 70 (2020) [19] in Turkey, 55 (2007) [16] and 7 (2020) [19] in United Arab Emirates, 5.9 (1982-1984) [21] and 15 (2020) [19] in Libya, 4 (1990-2000) [22] and 16 (2020) [19] in Oman, 59 (2020) [19] in Egypt, 12 (2020) [19] in Iraq, 40 (2020) [19] in Syria, and 12/100,000 (1985) [23] in Tunisia. In addition, study of demographic characteristics of MS in two ethnic groups, Persian and Arabic, showed that incidence and prevalence were higher in Persians [24,25]. This higher rates of Persian than other ethnicities were also observed in Parsis (people originated from Persian) in India [26]. Considering the recent trends, the hypothesis of Kurtzke has been rejected [27-29].

Analyses of studies on MS epidemiology have found an almost universal increase in prevalence and incidence of MS over time [4]. The prevalence and incidence rates of MS, as an autoimmune disease with unknown factor [1], have had an upward trend in many countries including Iran in recent years [1]. In addition, the prevalence of MS in Southeastern Iran was in the intermediate range; however, recent studies showed increasing disease incidence [30]. This article is going to update information about MS in Iran with focus on the potential association with air pollution.

## 2. Multiple sclerosis in Iran

Global burden of disease (GBD) 2010 study report has suggested that disability-adjusted life years (DALYs) lost due to MS has increased from 1990 to 2010 for both genders and all age groups in Iran [4]. In addition, ranks of DALYs and death attributable to MS in Iranian increased from rank of 122 to 105 and 90 to 74, respectively, in the period of 1990-2010 [4]. In addition, several studies suggested that MS incidence and prevalence have dramatically increased in Tehran over the past two decades; and the health impact of air pollution in Tehran underscores the attention to a possible association to this environmental risk factor [4]. The prevalence rate of MS in Torbat-e Heydarieh has also been increased from 1982 to 2016 [31]. The positive family history of MS, relapsing remitting, primary progressive, secondary progressive, and relapsing progressive prevalence in Iran was estimated about 8.9%, 77.1%, 6.2%, 9.5%, and 0.4%, respectively [32]. The prevalence of MS in Mazandaran Province, in north of Iran, has been increased from 20-60/100,000 in 2013 to 72.5/100,000 in 2018 [27,28].

Iran has traditionally been thought to be a low-risk area for MS; however, epidemiologic publications on MS either from the Isfahan MS center or other parts of Iran consistently have revealed that Iran now is intermediate to high-risk region [10]. Based on the published atlas of MS in Iran, it seems that there is a high-risk “belt” from northwest to southeast [10,33,34]. It is crucial to assess whether there is any association between soil pollution and such a high-risk belt [10]. In this regard, there is highly recommended to conduct a comprehensive evaluation study of the sources of water, soil, rock, sediment, plants, and crops in other provinces especially those known as high-risk regions in the epidemiologic studies focusing on the etiology of increased MS prevalence in Iran [10]. However, studies conducted in Iran are more sensitive to demographic changes (not affected by survival rate of MS) [35], unavailable data to diseased and emigrated patients or patients who are not registered in national MS registry [36], delay time between disease onset and diagnosis time [33].

Different factors such as the increased rate of smoking, lifestyle changes, modernization, and contact with toxic solvents might be considered as reasons for this sudden rise in the prevalence of MS in Iran; however, they are not definite causes [37]. The risk factors of the increased trend is unknown in Iran; therefore, an ecologic study has been conducted to evaluate the correlation between MS with urbanization, life style and industry in 2011, using three databases from national registry plans, considering provinces of Iran (n=31) as the subjects [1]. The results indicated an ascendant trend of MS during the recent years, with the change of MS incidence from 26.2/100,000 in 2006 to 44.5/100,000 in 2011 [1]. There was a direct significant correlation between the percentage of urbanization and the percentage of male smokers with the prevalence of MS in Iranian provinces [1]. The role of smoking was more highlighted due to its possible effect on the increasing risk and causing MS [1]. In addition, urbanization could be introduced as an augmentative factor; although, it is a combination of many complex factors [1]. Smoking might be considered as a potential factor to increase the risk of MS, urbanization can also be an augmentative factor; however, it is a combination of several complex factor [1]. Socioeconomic status (SES) in Iran is different from other countries; it seems that Iranian Provinces with a higher SES level have higher prevalence rates of MS [38].

Due to improving general health in Iran, which is consistent with “hygiene hypothesis”, it may be suggested the potential increase of the chance of autoimmune disorders like MS with lower exposure to external antigens [33]. In addition, more common Western lifestyle in Iranian population might be another explanation for increasing MS in Iran [39].

The declining pattern of MS incidence, especially after 2004, might be because of the time lag between disease onset and diagnosis as well as registering to national MS registry and less likely caused by real decline [40]. Moreover, higher prevalence and lower incidence rates may suggest relatively longer survivals of Iranian MS patients [36].

Based on the data of Iranian Ministry of Health, while the crude MS prevalence rate increased from 24.26/100,000 in 2006 to 54.51/100,000 in 2013, and the crude MS incidence increased from 3.77/100,000 in 2007 to 5.87/100,000 in 2013 [41]. The number of Iranian MS patients was 75,000 with the prevalence rate of 90/100,000 in 2020 [19]. Tehran, as the metropolitan of Iran, has the large number of epidemiologic studies on MS in recent years. The incidence rate of MS in Tehran has increased from 0.68 in 1989 to 5.7/100,000 in 2006 [33]. The total point prevalence of MS in Tehran was 115.94/100,000 in 2015 [42]. In a study on MS patients from 1991 to 2017, mean age of was onset around 28.64 years and point prevalence of 148.06/100,000 in 2017 [43]. Using a minimum data set (MDS) for MS patients in Tehran from October 2017 to March 2018, six main domains have been investigated consisting of patient identification, diagnosis, MS family history, disease course, medications, and disability status, to provide a standardized MDS to gather MS patients’ data for future researchers and policy-makers [44].

Within Iran in 2014, the highest and lowest prevalence was reported in Isfahan (93.06/100,000) and Golestan (18.0/100,000), respectively [13,33]. Concentration of advanced medical services in the city of Isfahan and its scientific authority in the center of Iran, can lead to patients referring to Isfahan from elsewhere, as a possible reason for the higher number of patients. In addition, Chahar Mahal and Bakhtiary (CMB), Fars, Tehran Provinces with the prevalence of 92.7, 77.3, and 74.3/100,000, respectively, are reported higher rates than before [3,45]. CMB province is a non-industrial and less-urbanized province and the potential risk factors such as air pollution and heavy metals are unlikely to be MS determinants there. In addition, due to low distance to Isfahan, many MS patients can register in the Isfahan MS center, without knowledge of MS centers in Isfahan and CMB Province.

Previous publications reported that Isfahan, a central province of Iran, could be considered as an area with a medium to high-risk of MS, especially in the young female population [13]. However, Isfahan Province is now very well-known because of its high prevalence of MS [46]. Its prevalence from less than 5/100,000 in 1980 [12], increased to 35.5/100,000 in the period of 2004-2005 [47], 43.8/100,000 and the incidence of 3.64/100,000 in the period of 2003-2006 [48], and prevalence of 73.3/100,000 and incidence of 9.1/100,000 in the period of 2003-2010 [49]. These evidences have highlighted Isfahan as a high-risk area considering its MS prevalence and incidence rates [13]. For instance, in a study in Isfahan, the high prevalence of MS has been approved, and Isfahan is considered as a medium- to high-risk area for MS [30]. Isfahan is the industrial heart of Iran and a well-studied area in terms of epidemiology of MS with a crude MS prevalence of 98.8 per 100,000 [10]. In addition, a retrospective study on MS patients in Isfahan in 2013, reported the MS prevalence of as high as 84.1/100,000, with the highest MS patients are located in the Isfahan rural area with 90% young patients (range: 20-50 years old) [13]. This study suggested that Isfahan and its rural provinces must be considered as two distinct regions, with medium-risk areas in Isfahan and high-risk areas in Isfahan rural areas [13]. Considering high air pollution, the presence of industrial factories located in the Isfahan rural areas could be an explanation and may lead to a need for future investigation in this direction [13]. Another reason might be associated with Zayandeh Rud river, where 80% of the extracted water is used for agriculture, especially its popular rice called Lenjan Rice [13]. The presence of different industries, history of elevated exposure to organic solvents, cosmetic colors, charisma of nitrophenol compounds in meat following nitrite curing and smoking, migration, and electrical power installers has been reported previously [13]. Moreover, existence of advanced medical services in Isfahan with high scientific authority in the center part of Iran can result in more referring of patients to Isfahan, as a potential reason for the bigger number of patients.

In a study to compare Armenian MS patients, the only Christian minority of Isfahan province, resided in Isfahan city from 2003 to 2014, with Persian MS patients, there was a significant difference between MS prevalence rates of two groups: 559.04 and 99.2/100,000 in Armenian and Persian patients, respectively [50]. However, the MS prevalence rate in Armenia was 3.75, 3.66, and 10/100,000 in 1981, 2007, and 2020, respectively [19,51,52]. The potential explanation might be due to exposure to the same environment, but with genetic and lifestyle differences between Persians and Armenian patients [50]. In addition, while sensory and visual symptoms were the most common presentations in Persians, the cerebellar sign and symptoms were more in Armenians [50].

## 3. Risk factors

Different causes can be considered for MS consist of infectious, social, and environmental. Most important infectious disease could be Epstein-Barr virus (EBV) infection [53]. Moreover, geographical latitude, stress, skin color, immigration, meals, Vitamin D intake, smoking, and occupational contact with toxins could also be considered; however, the substantial factors may include geographical latitude, Vitamin D, and immigration [54].

In addition to environmental factors, effects of genetic factors as well as mutuality of gene-environment interaction should also be considered [1]. Moreover, taking into account examined environmental risk factors in the past decades in Iran, EBV, ultraviolet (UV) light exposure/Vitamin D status, and smoking were the most important factors due to strength of supporting evidence [10]. The most important risk factors could be:

### 3.1. Gender

MS is an autoimmune disease of the CNS that affecting women more than men [30]. In 2020, 26% and 74% of Iranian MS patients were men and women, respectively [19]. In fact, MS is at least 3.55 times more common in women than in men, suggesting that sex hormones may also play a significant role on the susceptibility to MS [13]. In addition, a general increase in incidence of MS in women has been observed [4]. In 2013, out of 4536 registered MS patients in Isfahan, 3508 subjects were female and the rest (1028) were male, with a female:male ratio of 3.41:1 [55]. A hospital-based study in Isfahan reported that the number of MS women has increased from 2011 to 2013 [56]. Early-onset MS is not rare condition and among girls, it can be even observed in prepubertal period; although, there are some limitation such as inability of children to describe their symptoms or wide range of clinical presentations or various differential diagnosis [57].

### 3.2. Age

Majority of patients (about 90%) are diagnosed between the ages of 20 and 50 years, although MS can also occur in young children and older adults [13]. MS is indeed a chronic disease which is prevalent among young and middle-aged people [11]. In 2013, mean (±standard deviation) age at onset of MS in Iran was 33.1 (±9.5) years; with the overall age-adjusted prevalence rate per 100,000 increased to 85.8 compared to 71.6 in 2010; and the age-adjusted prevalence for females and males was 133 and 39.2/100,000, respectively [55]. For the population living in 2010, the 3-year incidence rate was 26.03 [45]. A hospital-based study in Isfahan revealed that from 2011 to 2013, the most common MS patients were at 20-39 years age group [56]. In a study on early MS onset in Isfahan, the mean age of neurological symptoms among MS patients with age of 16 and less was 14.7 (±1.8) year [57].

### 3.3. Early life exposure

Cognitive deficits in early life might be associated with prenatal and postnatal air pollutant exposures, which can affect on brain structural, volumetric, and metabolic changes in adolescence and even early adulthood with remarkable cognitive deficits which may impact negatively on the education, work, and social performance of the exposed individuals [58].

### 3.4. Family recurrence

In a population-based study from 1999 to 2015 in Tehran, the most part of relatives with MS were first-degree relatives, especially siblings and familial recurrence were correlated with relative type [42]. The MS familial recurrence was 13% (men) and 12.2% (women) [42] which is 2 times (6.12%), 4 times (3%), and 6 times (2%) higher than Brazil [59], Mexico [60] and Hungary [61], respectively, but less than Canada (19.8%) [6]. In addition, the mean onset age was 28.3 years which was similar to Malaysia (28.6), Japan (28.3), Taiwan (30.0), and South Korea (30.4), but less than India (38.3) and China (37.4) [62]. Moreover, the younger age of onset in MS familial recurrence was similar to the results of studies in Spain [63] and Argentina [64]. Furthermore, although MS prevalence is higher among women; familial recurrence was higher in men [42].

### 3.5. HLA-DRB1 polymorphism

The relationship of HLA-DRB1 polymorphism and MS susceptibility or resistance has been investigated through a systematic review and meta-analysis until June 2017 [39]. While HLA DRB1*15 was associated with increasing MS risk, there was an association of HLA-DRB1*03 and HLA-DRB1*04 with the disease phenotypic group, and protective roles of HLA-DRB1*07 and HLA-DRB1*11 phenotypes against MS incidence [39]. In a study in north of Iran, it has been suggested that low or no gene expression of major histocompatibility complex Class I chain-related gene B (MICB) has a beneficial effect to prevent of autoimmune response in the MS patients [65].

### 3.6. Vitamin D deficiency

The substantial role of Vitamin D deficiency in MS, especially among youngers and high school students, has been reported in Isfahan and Tehran [66,67]. It is believed that a major changing lifestyle trend during the past decades, spread of urbanization, indoor living, air pollution, changes in diet, widespread use of sun screens, avoiding sun exposure for fear of skin cancer, and concern about skin beauty can cause Vitamin D deficiency [3]. In addition, the relationship between month of birth and MS risk may be due to potential function of Vitamin D during pregnancy or early life period [68].

### 3.7. Environmental factors

The most substantial environmental factors include:

#### 3.7.1. Lifestyle

There is evidence about a higher risk of developing MS among populations with higher living standards; the potential explanation would be the potential weaker adaptation of the immune system to foreign agents in better economic status communities during childhood [69]. Urban life style and smoking could play substantial roles in MS risk increase; urbanization index in fact comprised multifactors that could affect on increase and decrease of diseases [1]. A study in Saudi Arabia reported that while drinking coffee, usage of fruits, and higher levels of sun exposure, especially in primary school and university can be considered as protective factors, consumption of fast food might be associated with the development of MS [70].

#### 3.7.2. Smoking

There is an association between smoking and the increased risk of MS (1.81 times more prevalent among smokers than non-smokers) [71]; but, it might be not a good explanation on higher MS in Iran, due to low prevalence of smoking [72]. However, there are some evidences about the more possible exposure with passive or second-hand smoking [73].

#### 3.7.3. Exposures with chemicals

Although there is no reliable evidence for the association between MS development and occupational or non-occupational exposures to chemicals [74]; there are some evidences that recommend the avoidance from toxic chemicals to prevent the development of MS [75], because access to toxic chemicals is too easy for Iranian people with no specific rules and regulations for the obtaining these substances, increasing the likelihood of developing MS due to exposure to chemicals [76,77].

#### 3.7.4. UV

Iran has been considered as a low-risk area for MS due to the inverse correlation between UV radiation and MS prevalence [30]. Therefore, birth month can play a major role in MS development, because the amount of winter sunlight could affect the prevalence of disease and high UV exposure can cause lower prevalence of MS [30]. Thus, Iranians born in April and May are more susceptible to MS due to low exposure to UV radiation [30]. A similar correlation has also been observed in Australia which suggested that the regional variation in MS prevalence may be predicted by regional UV radiation levels [30]. Moreover, it has been demonstrated that there is a negative association between mortality from MS and residential and occupational exposure to sunlight [30]. Furthermore, the potential reason for increased prevalence of MS among Iranian born in May might be due to maternal end-of-winter deficiencies in Vitamin D [30]. Thus, birth month could be considered as an important factor in MS prevalence and high exposure to UV radiation can reduce the risk of MS [30].

#### 3.7.5. Radon

Radon can also be considered as a potential risk factor for developing MS [78]. There are some areas of Iran such as Ramsar that Radon is naturally can be emitted from the soil; then, rocks and stones might absorb the emitted rays, including decorative stones, especially granites, which are commonly used in the buildings in Iran with no supervision or prior evaluation [79,80]. Therefore, more investigations are needed to fully understand the exact effect of Radon.

#### 3.7.6. Redox metals

The effects of redox-active metals (iron and copper) and redox-inactive metals (cadmium) on MS have been studied in recent decades [81]. While, the essential metals (iron and copper) can conduct neurotransmission, regulate the gene expression, maintain cell structure, and involved in homeostasis of antioxidant response, cadmium can influence MS-related attention, memory, motor speed, and its turnover may affect polyneuropathy [81]. In addition, while remarkable reduction of iron and elevation of oxidative stress has been found in MS patients [82]; copper’s role in MS could be associated with the excessive copper and subsequent oxidative damage [83]. Moreover, high concentration of cadmium among MS patients may be related to ­glutation-S-transferase (an oxidative stress associated with gene and metals biotransformation regulator) [84]. Environmental factors can be considered as the key reasons for increased cadmium level in the human body; this excess cadmium contents has been found in MS patients living in industrial areas or in foods such as corn, rice, and wheat in industrial areas [85]. The long-term exposure to air pollutions (including cadmium) can also have a substantial role in increasing MS risk [4].

#### 3.7.7. Pollutions

A significant etiologic role has been suggested for exposure to toxic elements on MS risk [10]. As a result of industrial activity, land application of bio solids from wastewater treatment and various by-products, Isfahan Province soil could have high levels of potential toxic heavy metals [10]. However, few epidemiological studies explored the effect of soil pollution on MS risk in Asia, especially Iran [10]. Among all pollutants, heavy metals and air pollutions will be discussed in this article.

#### 3.7.8. Heavy metals

There are few studies assessing the incidence of MS with soil heavy metal concentrations [86]. In addition, there is a controversy for the potential association of heavy metal soil content and MS; for example, while in Taiwan, it has been reported that exposure to lead in soil was positively associated with MS incidence [86] and lead content of soil in Henribourg was significantly higher than controlled areas [87]; another study revealed that MS did not appear to cluster around the lead smelter in Jefferson County, Missouri, USA [88]. Moreover, evidence of a case study in Italy suggested that removing heavy metals from the blood by EDTA chelating could improve the quality of life among MS patients [89].

In a Taiwan study conducted on new MS patients in 1997-2008, soil heavy metal factors records were consist of lead, cadmium, chromium, copper, arsenic, mercury, nickel, and zinc from 1986 to 2002 [86]. The association of soil heavy metals and age- and gender-standardized incidence ratios for townships was controlled for the sunlight exposure hours, smoking prevalence, and spatial autocorrelation [86]. The lead concentration in the soil was positively correlated with the township incidence; however, the arsenic concentration in soil negatively correlated with the township incidence and when found together, they have controlled each other [86]. In addition, while the exposure to lead in soil was positively associated with MS in men, exposure to arsenic in soil was negatively associated with MS among women [86]. Moreover, the workplace exposures to heavy metals may confound with the soil exposures; as well as migrations which can also produce bias due to misclassification of residences [86]. Furthermore, in epidemiological studies, the time of first symptom onset is the traditional starting point for any disease including MS and exposures at or before the time of first symptom onset are not known [86].

To better understanding of high MS rates in Iran, a study assessed the association of soil heavy metals with MS distribution in Isfahan [10]. Absorbable lead and absorbable cadmium concentrations, respectively, had a positive and negative correlation with the prevalence of MS; however, the role of potential confounders should be taken into account to interpret the data [10]. There are no reliable soil data from the entire Isfahan province, although it has been attempted to cover more than two-thirds of patients with MS over the sampling area [10]. As there is scarcity of data on the serum level of heavy metals in patients with MS, it should be confirmed by further investigations and measurement of serum levels of heavy metals in patients with MS and their matched healthy controls [10].

To measure blood lead and cadmium concentration and potential association with smoking habit, a case–control study was conducted in Tehran using patients registered from 2013 to 2014 [90]. While there was no significant difference between MS patients and healthy controls regarding lead, there was a significant difference in cadmium concentration between cases and controls with elevated cadmium blood level in MS patients; in addition, a significant association was found between cigarette smoking and concentrations of these two metals [90].

#### 3.7.9. Air pollution

Although in a study in USA, the exposure to PM air pollution was not related to MS risk [91], recent studies have shown that fine PM (PM 2.5) and three pollutants (SO_2_, CO, and lead) were statistically associated with higher pediatric MS [92]. However, depending on potential health effects and regulations in the different countries, only some air pollutants in the atmosphere are monitored, for example, particulate matter (PM), lead, sulfur dioxide, carbon monoxide, ozone, and nitrogen dioxide [58]. The other air pollutants which may be referred as hazardous pollutants should also be measured due to their highly neurotoxic chemicals are included metals (lead, cadmium, cobalt, iron, manganese, mercury, and arsenic) and volatile organic compounds (toluene, formaldehyde, benzene, tri- and tetrachloroethylene, and polycyclic aromatic hydrocarbons PAHs) [93]. In a study in Tehran, a significant difference was observed in exposure to PM_10_, SO_2_, NO_2_, and NOx in MS cases compared with controls; which confirm that the long-term exposure to air pollutants can be act as an environmental risk factor in MS [4]. There is evidence to confirm the potential effect of PM_10_ on the risk of relapse in MS patients, might be through oxidative stress mechanisms [94]. The exposure to air pollutants may initiate destructive mechanisms inducing reduction of immunological self-tolerance, inflammatory-oxidative cascades, and neurodegeneration leading to brain autoimmunity, might be considered as a potential hypothesis [95].

The knowledge of air pollution and its quality over periods and the process of air pollutants’ changes in various locations, and detection of high risk places can play a substantial and efficient role in managing of the urban health management and land use policymaking [96]. In fact, the air quality markers and the pollutants are known as immune reactors in general [97]. A study conducted to analysis the air quality of Tehran, for SO_2_, from 1995 to 2002 [98]. Measurements were collected from the seven main monitoring stations in various locations of the city [98]. Annual, seasonal, and diurnal variations were studied which yearly variation did not show any specific trend initially but a little upward trend in the recent years. The pick of SO_2_ concentration was seen during 6-12 h and during the winter season especially in January [98]. This study was assessed the effect of the meteorological parameters on the concentration of pollutant as well [98]. In addition, temperature, dew point, wind direction, the wind velocity, relative humidity, and rainfall were considered as independent variables [98]. The study revealed that the wind speed, daily temperature, and humidity have had reverse effect on the SO_2_ concentration [98]. Surface roughness, geostrophic winds, mixing height of the atmosphere, emission rate of the pollutant sources, and background pollutant concentration have also considered as the input parameters [98]. Findings showed that the air quality was different in different areas of Tehran and the pollutants moved to other areas by wind or other factors [97]. This transfer of the pollutants changed the concentration levels in various locations and such effects were difficult to model [97]. The most typical model in one of the explained locations was demonstrated using a monthly mean of NO_2_ concentration [97].

A significant seasonal difference in the spatial variation of nitrogen oxides in Tehran, especially NO_2_ was also found in a study [99]. Moreover, in January 2012, a study in Tehran, on CO and PM_10_ reported the possibility of occurring a pollutant in different locations, based on location information [96]. In another study in Tehran, among MS patients diagnosis with disease onset during 2003-2013, the spatial distribution of prevalent MS cases and their association with the spatial patterns of air pollution was assessed [4]. The location of MS patients was then geo-referenced within Tehran metropolis by geographic information system bureau of Iran’s post office based on their phone numbers. In addition, a cluster analysis was applied using the average nearest neighbor index and quadrat analysis [4]. Moreover, the long-term exposures of MS patients to nitrogen oxide (NO), nitrogen dioxide (NO_2_), nitrogen oxides (NOx), particulate matter (PM_10_), and sulfur dioxide (SO_2_) were estimated using the previously developed land use regression (LUR) models [4]. The study showed a clustered pattern of prevalent MS cases in Tehran. A significant difference in exposure to SO_2_, NO_2_, PM_10_, and NOx was observed in MS cases compared with controls [4]. The study revealed the potential role of long-term exposure to air pollutants as an important environmental risk factor in MS [4]. The findings were (1) the MS patients in Tehran follow a clustered pattern and (2) there is a statistically significant spatial correlation between the clustering of MS patients and the concentration patterns of SO_2_, NO_2_, PM_10_, and NOx [4].

Similarly, to quantify effects of health impacts of short-term exposure to specific air pollutants including PM_10_, SO_2_, and NO_2_, on some health consequences such as cardiovascular and respiratory diseases (especially chronic obstructive pulmonary disease (COPD)), one study in Shiraz, south of Iran, has been conducted [100]. This study was estimated excess hospitalization cases related to cardiovascular diseases, respiratory diseases (especially among elderly), and COPDs due to short-term exposure to air pollutants [100]. The estimation of number of excess hospitalizations due to respiratory diseases as a result of short-term exposure to SO_2_ and NO_2_ was categorized into three age-groups including<15, 15-65, and≥65 years old [100]. The study reported that air quality could affect daily hospital admissions dramatically [100].

## 4. Discussion

There is a close association between MS prevalence and modernization, lifestyle changes, industrial growth, and urbanization in Iran [37]. MS prevalence in Iran had a significantly upward trend in past years and this can be related to many potential factors such as urban life and unhealthy lifestyle [1]. Iran has a high MS prevalence rate and the highest prevalence rate in Asia and the Middle East, with no significant latitudinal difference was seen from the south to the north of Iran, no significant difference was observed in the prevalence of MS between the areas with low sun exposure (north of Iran) and those with high sun exposure (south of Iran) and genetic susceptibility and ethnicity might be more important than environmental factors in the susceptibility of the Iranians to MS [41]. It seems that conducting studies with focus on other effective factors such as education, social class, income, contact with air pollution and chemical solutions, and passive- or second hand smoking would be necessary [1].

MS is a complex disease, with several potential environmental factors which are thought to act together in genetically susceptible individuals to trigger the pathophysiologic process [10]. Environmental factors such as sunlight, through UV radiation, can have a substantial role in MS prevalence [30]. MS is more common in mid latitude temperate climatic areas, such as the northern United States, Southern Canada, and Northern Europe [30]. It has been shown that there is a low risk of MS in the hot, moist, and extremely dry equatorial zone [30].

The estimates from the GBD study indicated that approximately 87% of the global population is exposed to ambient concentrations of fine particulate matter (PM_2.5_) that does not meet the guideline values set by the World Health Organization [101]. This estimate is even higher when it is restricted to the populations of low- and middle-income countries; in addition, air pollution is one of six modifiable risk factors associated with more than 5% of the GBD, as measured by DALYs lost [99]. This burden is also reflected in Iran, where the estimates suggest that approximately 7% of total DALYs can be attributable to air pollution, which is 10 times greater than the DALYs attributable to HIV/AIDS and tuberculosis combined [99]. However, the burden of air pollution might be substantially underestimated due to two important reasons: (1) Most of the exposure-response estimates are from high-income countries and (2) the burden might not be fully captured by PM2.5 and ozone, which were the only indicators used in the GBD analyses [99]. Furthermore, emerging evidence suggests that air pollution is associated with many other chronic diseases not yet included in the GBD assessment, such as diabetes, metabolic syndrome, atherosclerosis, high blood pressure, and possibly with neurodegenerative diseases such as MS, vascular dementia and Alzheimer’s disease [99].

Air pollution can be considered as a complex mixture of gases and particles which is associated with a wide range of health consequences [99]. Outdoor air pollutants are indeed a combination of dangerous gases (e.g., ozone), organic compositions, and PMs [97]. Pollutants such as sulfur dioxide, aerosols and carbon monoxide, known to be the most important factors related to lung, heart, and vascular diseases, have underlined public health, making remarkable expenses for diseases resulting from these pollutants per year [96]. In addition, different studies have illustrated that air pollution can result in neurological disorders [97]. Moreover, the knowledge about MS relapse risk factors is limited; however, air pollutants might be regarded as a risk factor for MS relapse [97]. Furthermore, according to various evidences, some air quality markers such as NO_2_ may be linked to MS relapse rate; however, the MS relapse rates were not correlated with CO levels; however, PM_10_ was correlated with MS attacks in non-users of beta-interferon [97]. It should be taken into account that although the potential role of long-term exposure to hazardous environmental factors in the MS pathogenesis is not well established; however, it might be relevant in particular in the countries with continued environmental degradation and high MS incidence [4]. In a 8-year study of MS patients in Isfahan, the important effect of air pollution (measured by air quality index) on severity and remission of MS disease has been found [102]. In addition, 71% of MS patients were depressed, which might be due to direct effect of air pollution on depression resulting in direct or indirect effect on severity of MS [102].

Urban air pollution modeling is a complex forecasting problem and one or two assumptions are not sufficient to cope with it [98]. It is necessary that reliable models of atmospheric dispersion are available, if dispersion model must play a useful role in urban planning or in devising pollution control strategies [98]. This developed dispersion model can be used for Tehran city [98]. The advantage of this model is simple application because it does not contain of complex equation [98]. It can be used easily to predict and control the status of SO_2_ in the city [98]. By considering this model, the concentration of pollutants could be studied with present and absence of source, such as urban centers and outskirts. Moreover, there are some annual and seasonal LUR models for ambient oxides of nitrogen (NO_2_, NO, and NOX) in Tehran using 2010 data from 23 fixed monitoring stations through developing a systematic algorithm for spatial modeling [99]. The most predictive variables were: Distance to primary schools, green spaces, official areas, bridges, and distance to the traffic access control zone and slope [99]. The annual average concentrations of all pollutants were high compared to those reported for megacities in Asia. At 1000 randomly selected locations, the correlations between cooler and warmer season estimates were 0.30 for NO_2_, 0.64 for NO, and 0.58 for NOX. In addition, seasonal differences in spatial patterns of pollution were likely driven by differences in source contributions and meteorology [99]. The models have provided a basis for understanding long-term exposures and chronic health effects of air pollution in big cities like Tehran [99].

The related issues to trade liberalization and its impact on the environmental quality have become important since late 1996 [103]. There are also some effects of inter-industrial trade on the Iran’s air pollution [103]. The effect of inter-industrial trade on the environment was studied in two ways: (1) Direct effect on the environment and (2) an indirect effect through growth and the effect of growth on the environment [103]. The model has been estimated using the panel data method for Iran’s various industrial sectors over 1980-2014 [103]. The results revealed that inter-industrial trade has positive effect on Iran’s air pollution and trade liberalization has led to the reduction of environmental quality [103]. In fact, many economic activities of different industrial sections have destroyed the environment at raw material extraction step, utilizing renewable resources or waste, and pollution creation [103]. Iran has indeed comparative advantage in producing dirty industries [103]. Furthermore, pollution haven hypothesis is accepted in Iran industries, because lax environmental policy increases trade and attraction of foreign investments for more production and exportation of dirty industry [103]. In general, obtained results revealed the necessity of some measures to be taken by which different industrial sections are related to pollutant emissions; therefore, it is required to get environmental certificates; however, due to high costs of such standards, it is necessary for government to adopt supportive policy or increase the firms’ incentives for this purpose [103].

## 5. Conclusion

MS prevalence across Iran is geographically different, from 5.3 to 74.3/100,000 [3]. In 2020, the prevalence rate of MS was 90/100,000 in Iran [19]. This difference might be explained by different methodology, underestimation of the real incidence, referral bias, and various climates as well as great variations in diet and environmental exposures to many factors, both traditional and modern risk factors [3]. Air pollution can be the main risk factor of MS in Isfahan and Tehran Provinces, but not in CMB province. Future studies are needed to find out this debate.

The future of Iran starts today; tomorrow will be too late, then some suggestions such as increasing public transfer facility, extending network of the metro in the big cities, banning of old technology of vehicles and industries, encouraging the industries to obey the standards of air quality, controlling traffic, controlling automobile factory for upgrading technology and using up-to-date standards to design engine and protecting the environment against air pollution can be adopted by the local government for enforcing the existing laws for environmental conservations [98].
